# Comparing the effectiveness of narrative therapy and EMDR-GTEP protocols in the treatment of post-traumatic stress in children exposed to humanitarian crises

**DOI:** 10.3389/frcha.2024.1320688

**Published:** 2024-05-24

**Authors:** Elisabetta Dozio, Cécile Bizouerne, Valdes Wamba, Ninon Atienza

**Affiliations:** ^1^ Mental Health and Psychosocial Support, Action contre la Faim, Paris, France; ^2^ INSERM U1018 Centre de Recherche en Épidémiologie et Santé des Populations (CESP), Villejuif, France; ^3^ Independent Researcher, Paris, France

**Keywords:** PTSD, children, paraprofessional, EMDR, G-TEP, CBT, TF-CBT

## Abstract

**Background:**

The mental health of children living in humanitarian crisis situations is a major issue. Post-traumatic stress disorder (PTSD) causes great psychological suffering and has negative consequences on children's development. The aim of the study was to analyze retrospective data collected in a mental health and psychosocial support program for children in the Central African Republic, and to compare results of two trauma-focused treatment interventions: the narrative protocol Action contre la Faim (ACF)/KONO; and the EMDR-based Group Trauma Episode Protocol (G-TEP). Both protocols are proposed in a group setting and led by paraprofessionals.

**Methods:**

In the program, 884 children attended a psychoeducation session and after that, 661 children (290 for ACF/KONO and 371 for G-TEP) benefited from all treatment sessions. PTSD was measured by the Children's Revised Impact of Event Scale (CRIES-8). General distress was measured by the Child Psychosocial Distress Screener (CPDS). Data were collected before and after treatment, and measured 5 months after the end of treatment for 185 children.

**Results:**

Participants in the ACF/KONO group show a significant reduction on CRIES-8 (*t* = 44.8; *p* < 0.001, effect size = 2.63) and CPDS (*t* = 38.2; *p* < 0.001, effect size = 2.24). Participants to the G-TEP protocol show a significant effect with reduced scores on the CRIES-8 (*t* = 49.2; *p* < 0.001, effect size = 2.55) and CPDS (*t* = 57.2; *p* < 0.001, effect size = 2.97). A Student's *t*-test comparing the ACF/KONO and G-TEP groups shows no significant difference between the two types of treatment between pre- and post-treatment CRIES-8 scores (*t* = 1.744; *p* = 0.514, effect size = 0.040) and CPDS scores (*t* = 1.688; *p* = 0.092, effect size = 0.323). An analysis of the follow-up data for the 185 children shows that the effects of both protocols are maintained over time with mean scores after treatment and follow-up below the clinical cut-off for both CPDS (<8) and CRIES-8 (<17).

**Conclusions:**

Both protocols have been shown to be effective in reducing traumatic symptoms in children exposed to conflict; they can be conducted by paraprofessionals and used in humanitarian crisis situations.

## Introduction

More than 1 in 6 children and adolescents worldwide (468 million in total) live in areas affected by armed conflict (1). In these contexts, children are exposed to all kinds of violence, abuse, exploitation, and neglect; they may die or be injured, experience malnutrition and other illnesses, lose or be separated from their families and loved ones, and have poor access to basic services. These factors have a major impact on their survival, growth, and development (2, 3). It is estimated that approximately 10%–20% of children worldwide experience mental disorders (4–6). In war, these disorders are more common and include post-traumatic stress disorder (PTSD) and post-traumatic stress symptoms, behavioral and emotional symptoms, sleep problems, disturbed play, and psychosomatic symptoms, anxiety disorders, and depression (7–10). The effects on mental health depend on the child's age, caregivers’ emotional situation and their capacity to take care, daily safety, and protection (10, 11). PTSD has devastating consequences for children, alters the architecture of their brain (12), and puts their development at risk, as well as their ability to concentrate, keep up with schooling, and build appropriate and ongoing social and emotional relationships. Recognition, prevention, and treatment of PTSD in conflict zones are still largely inadequate (13–15). Data on psychological interventions for children in war situations are few and of insufficient quality to demonstrate a beneficial effect of therapies on the reduction of PTSD symptoms (16). This public health problem needs to be addressed for children so that they can enjoy a psycho-emotionally healthy future. Internationally, the recommended treatments for PTSD are Cognitive Behavioral Therapy (CBT) and Eye Movement Desensitization and Reprocessing (EMDR) (17–21). CBT is a form of brief therapy that aims to modify negative thoughts, emotions, and reactions. Adapted to the treatment of trauma, trauma-focused CBT (TF-CBT) includes psychoeducation, exposure to traumatic memories, and cognitive reprocessing on the symptoms and effects of PTSD. EMDR is a therapy developed by Francine Shapiro (22–24), based on the Adaptative Information Processing (AIP) model. The therapy is in eight phases and includes bilateral stimulation, and aims to facilitate access, process traumatic memories or adverse experiences (25) to bring an adaptative resolution. Studies comparing CBT and EMDR with children and adolescents show no difference in terms of efficacy (26–28) or a better efficacy of EMDR (29) but with a too limited number of EMDR studies. This explains why recommendations focus first on CBT and then on EMDR if CBT does not work (30, 31). These two therapies are often proposed in an individual setting and conducted by psychologists after extensive training. In most emergencies, there are no, or not enough, trained specialized professionals. Task shifting might be an option to scale up access to mental healthcare in addition to brief intervention protocols (32–36), even if more research is required to evaluate their effectiveness (37–39). Therefore, it is important to evaluate the effectiveness of brief (1) intervention protocols (2) in groups, to treat PTSD in children (3) in emergencies and (4) in countries where the number of mental health professionals is very low and to demonstrate that treatments for PTSD in children do exist, are feasible in emergency contexts, and that they allow for wide coverage since they are part of a public health approach.

The Central African Republic (CAR) is an example of emergency context, where mental health and psychosocial problems are high but with a limited capacity in mental health professionals. A decade after the military-political crisis of 2013, CAR has yet to benefit from peace and sustainable development. According to the annual report of the United Nations Office for the Coordination of Humanitarian Affairs (40), almost 265,000 people have been directly affected by conflict-related shocks. The north-western regions (Ouham, Ouham-Pendé) recorded the highest number of people affected, representing 45% of the population affected by violence. Documented violations include summary executions, rape, torture and cruel, inhuman, and degrading treatment, arbitrary arrest and detention, kidnapping, destruction or looting of property, and the recruitment and use of children by national forces and armed groups. Some civilians were targeted because of their ethnicity or religion.

The Humanitarian Needs Overview (HNO 2022) (40) reveals that children have been subjected to physical or psychological violence during forced displacement and that, because of these incidents, traumatized children have lost their zest for life as well as their sleep. Nationally, almost one in two households (44%) has at least one child who has shown symptoms of a mental disorder in the last 2 weeks, particularly sleep disturbances, sadness, loss of appetite, and unexplained tiredness. This feeling of distress is more prevalent among children living in the high-violence areas of the north-western, central, and south-eastern prefectures. The psychological and social consequences of the crisis in CAR are serious and risk compromising the mental health and psychosocial wellbeing of children and adolescents in the long term. The HNO 2022 reports changes in children's behavior since 2021. The most frequently cited are negative coping behaviors, such as aggression (61%), refusal to go to school (54%), violence against young children (41%), and an increase in high-risk sexual behavior. Children and adolescents affected by the conflict need appropriate psychological support to help them regain a sense of security and give them the opportunity to overcome traumatic experiences and develop skills to cope with future crises, regulate their emotions, and establish and maintain positive relationships (41). However, resources dedicated to mental health are scarce and insufficient to fill the considerable gaps that exist in CAR (42). The psychiatric hospital in Bangui remains the only specialized structure offering mental healthcare. In terms of governance, a national mental health policy was drawn up in 2011, but it is experiencing implementation difficulties. This has led to an extremely limited mental health support capacity at national level.

In this context, the non-governmental organization Action contre la Faim (ACF) has been running mental health and psychosocial support programs in CAR since 2008. These projects offer psychosocial support to people in distress, including children. In particular, during 2016, children aged 6–17 years, who had been directly exposed to potentially traumatic events, took part in group psychological treatment according to the narrative protocol: ACF/KONO (43). The aim of this psychological support was to strengthen individual and collective resilience to cope with new living conditions, guarantee psychosocial wellbeing, and support children's healthy development, reducing symptoms of post-traumatic stress.

This protocol has been shown to be effective in reducing traumatic symptoms and psychic distress in children, although it has a number of limitations that may limit its use in emergency contexts. The protocol consists of five sessions. However, in conflict and highly insecure situations, it is not always possible to guarantee this continuity of care, either because the teams and/or participants cannot return to the sites because it is too dangerous, or because the populations are displaced and flee or return to their homes. Another specific feature of the ACF/KONO protocol is that people are asked to narrate their trauma and/or difficult events. This can be both a factor of cohesion and potential identification within the group as well as a point of support for overcoming traumas. On the other hand, it can sometimes make the process complicated, either because of the PTSD itself or because these events can lead to shame and limit sharing within the group. The final element concerns the risk of vicarious traumatization, as participants and teams hear each other's traumatic experiences. These factors led us to evaluate the feasibility and effectiveness of other intervention protocols for dealing with trauma.

As part of this type of project, the ACF team has decided, in 2022, to introduce a new approach based and adapted from EMDR therapy, which has proved effective in other contexts: the Group Trauma Episode Protocol (G-TEP) developed by Shapiro and Laub (44).

Before extending the use of this new protocol, it was fundamental to analyze the results by comparing them with the ACF/KONO protocol that had already demonstrated its effectiveness.

The overall objective of this study was to contribute to improving the management of symptoms of PTSD in CAR by comparing the results of two trauma-focused treatment interventions for children to assess the impact of therapeutic approaches adapted to trauma management in humanitarian contexts. In addition, the study also had the specific objective of assessing the maintenance of improvement in wellbeing and reduction in traumatic symptoms over time.

The results will make it possible to adapt trauma management protocols that have a positive impact on improving children's wellbeing, and to observe any differences between protocols to make better choices according to humanitarian situations and constraints.

## Materials and methods

### Participants

#### Inclusion criteria

The population participating in the program consisted of children exposed to traumatic events due to the ongoing conflict in the country. The program was carried out in the prefectures of Nana-Mambéré, Ouham, Ouham Pendé, and Mambéré Kadei. Children aged 6–17 years who had been exposed to traumatic events and had scores of 17 or more on the Children's Revised Impact of Event Scale (CRIES-8) were eligible for the study.

#### Exclusion criteria

Children with psychiatric disorders assessed by the ACF psychologist were excluded from the program and referred to specialized services for appropriate care. When the mental health specialists were not present in the area, advocacy was carried out with the Protection and Health Clusters and the mental health working group.

### Procedure

Children were recruited in localities where the ACF team had been deployed after a critical incident. The methodology consisted of starting with a psychoeducation session on the normalization of traumatic symptoms organized within these communities. Children were free to attend this session. After this session, the presence of PTSD was assessed using the CRIES-8 for children willing to participate in the program. Children with scores of 17 or higher were included in the program if they wished and randomly assigned to the ACF/KONO or G-TEP therapeutic group.

**Figure 1:**
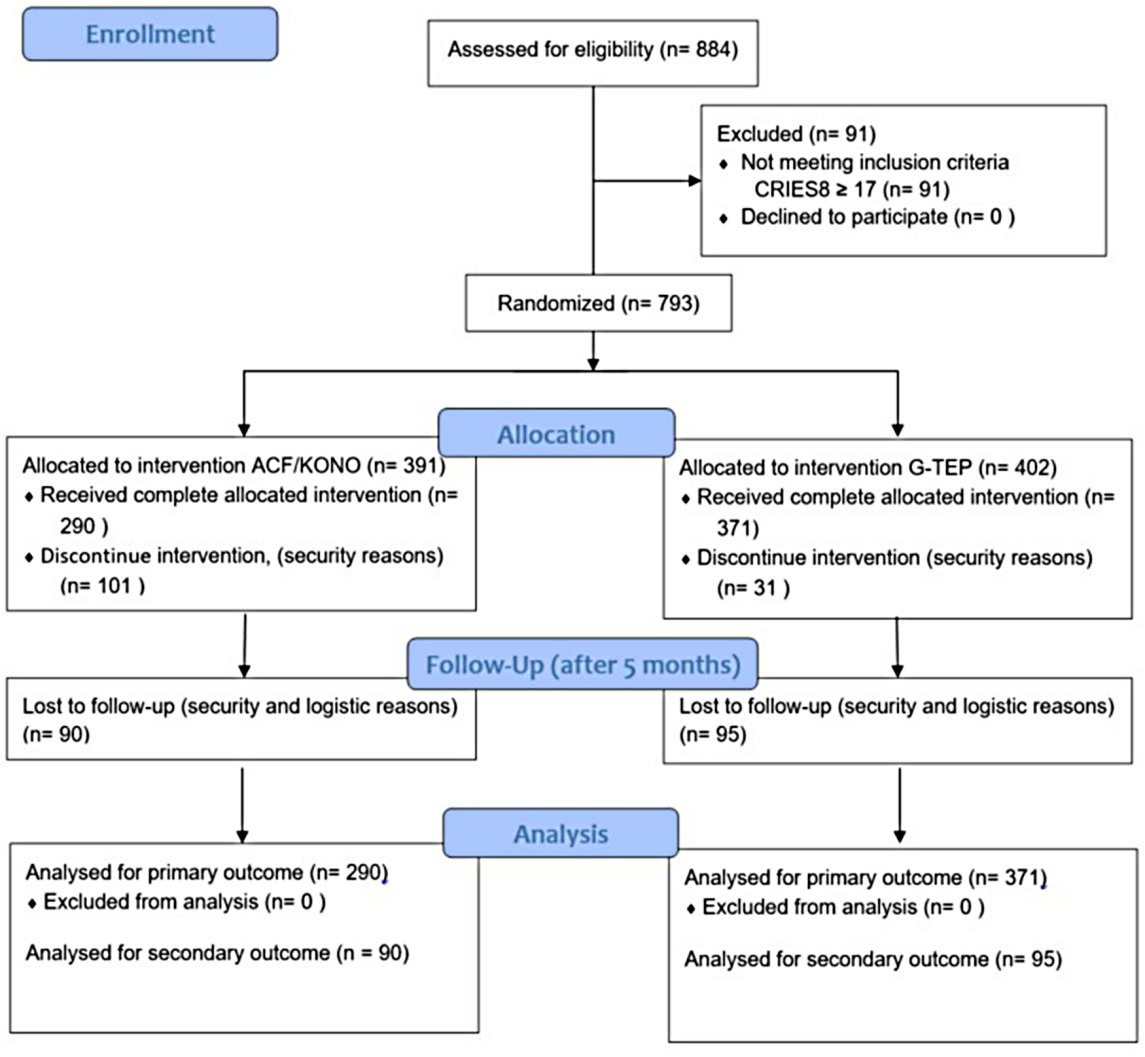
Program design and flow of participants.

In total, 884 children have participated in psychoeducation and 793 have been invited to take part in the program (Figure 1). A total of 402 children participated in the ACF/KONO protocol and 391 in the G-TEP protocol. The intervention consisted of five bi-weekly sessions for each protocol. Data were collected using a standardized questionnaire that included demographic data on the children (gender, age, etc.). Psychometric data were collected at three time points: at admission, at the end of treatment, and 5 months after the end of treatment. For security reasons, it was not possible to return to all the intervention areas. We were only able to carry out the follow-up in two areas and reach a sample of 185 children to repeat the psychometric scales and measure the effect of the two therapies over time.

The intervention followed the principles of the Declaration of Helsinki and international guidelines on ethics in clinical research (Council for International Organizations of Medical Sciences-CIOMS, 2009). Before the psychoeducation session, before data collection, parents and children were given full information about the program. Parents gave written informed consent for their child to participate. Participation was voluntary and children could withdraw at any time without giving any reason for their absence. The data collected were completely anonymous. The databases did not contain any elements that might allow participants to be identified. This is a normal procedure in emergency settings to protect the information of individuals and ensure confidentiality and their security.

The study consisted of a secondary analysis of anonymized data collected as part of routine monitoring and evaluation activities conducted by ACF for its programs in 2022. Given the exclusive use of de-identified secondary programmatic data, this study is not considered to be research involving human subjects, in accordance with the CNIL's Deliberation No. 2018-155 of 3 May 2018 approving the reference methodology relating to the processing of personal data implemented in the context of research not involving the human person, studies, and evaluations in the field of health (MR-004).

### Protocol ACF/KONO

The trauma-focused narrative protocol “KONO” has been developed and implemented in several humanitarian contexts by Action Contre la Faim. A study in the Central African Republic validated its effectiveness on 674 children aged 6–16 years, showing a significant reduction in symptoms of trauma (43). The sessions last 90–120 min and a different theme is addressed each time (loss and bereavement, traumatic events, the future). The children are invited to share their fears, negative emotions, and resources, and to talk about what they have been through and how they imagine their future. The psychosocial worker accompanies the process, providing emotional support to the participants and helping them to tell their stories with the help of the group. The facilitator provides psychoeducation and relaxation and stress management techniques. Children are also invited to use drawings at each session (45).

### Protocol G-TEP

The G-TEP, developed by Elan Shapiro (46), is a simplified adaptation of the recent traumatic event protocol (R-TEP) (44) for adults, children, and adolescents who have had recent or past traumatic experiences with effects in the present. It is a group-based intervention for treating a traumatic episode to reduce traumatic stress, promote adaptive processing, strengthen resilience, and prevent post-traumatic complications (47). Each G-TEP session is the same, unlike the ACF/KONO protocol. The protocol includes connection to past, present, and future resources that can be shared in a group, exposure to traumatic memories and alternating bilateral stimulations. The effectiveness of G-TEP has been demonstrated after two sessions (48). G-TEP is used by mental health specialists who have been trained in EMDR, which greatly limits the possibility of deploying it on a large scale in low- and middle-income countries (LMICs) or in conflict zones where mental health professionals are rare and those trained in EMDR are even rarer. The G-TEP is a simplified, comprehensive protocol, based on a G-TEP sheet, filled in by the participant as the session progresses, with distancing via drawing, and which is implemented in a group setting. As such, it could offer an alternative to the “classic” EMDR protocol through the training and supervision of paraprofessionals by G-TEP-certified trainers. Recent research has shown the effectiveness of G-TEP used by paraprofessionals in reducing traumatic symptoms in adults in conflict settings (49).^1^ The aim of this research was to verify the validity of the strategy for scaling up this EMDR-adapted protocol for children.

### Profile and training of the team

The team consisted of 10 psychosocial workers, 2 supervisors, 1 project manager (expatriate clinical psychologist), and his deputy. The psychosocial workers and the supervisors were secondary school graduates with various university qualifications (law, health assistant, nursing assistant, management sciences, geography, social sciences technician, etc.), seven of whom had at least 3 years of experience in psychosocial and psychological support activities at ACF, the others between 18 months and 2 years. All had received training in psycho-trauma and the protocols used (G-TEP, ACF/KONO). They carried out the psychological treatment under the supervision of two supervisors who provided ongoing technical support in setting up the treatment groups. The Deputy Program Manager (who holds a master’s degree in public law and has extensive experience in mental health and psychosocial support programs) was responsible for planning and organizing activities in the field. The clinical psychologist was responsible for the technical quality of the interventions, training the teams, and analyzing the quality of the data collected. The entire team was regularly supervised remotely and during field visits by a clinical psychologist trained in EMDR and the G-TEP group protocol.

### Measurements

Two scales were used in this program.

The CRIES-8 (50) is an eight-item scale used to screen children at high risk of PTSD. It is adapted by the Children and War Foundation from the Impact of Event Scale (51) that assesses subjective distress caused by traumatic events in adults. Items are rated on a 4-point scale (none = 0, rarely = 1, sometimes = 3, and a lot = 5), according to frequency over the past week, and in relation to a specific traumatic event. The total score is in the range of 0–40, with higher scores indicating more severe post-traumatic stress symptoms.

CRIES-8 psychometric properties were assessed in two studies. One involved 87 children who survived a cruise ship sinking (52), while the other included 170 children attending a hospital emergency department after road traffic accidents or sporting injuries (53). Both studies supported the reliability and validity of the CRIES-8 as a screening tool for PTSD. Regarding reliability and validity, the eight-item version (52) showed a strong correlation (*r* = +0.95, *p* < 0.001) with the total score of the 15-item version from which it originated. In a study of 87 shipwreck survivors, those diagnosed with PTSD according to Diagnostic and Statistical Manual (DSM) criteria scored significantly higher (26.0) on the eight-item version compared to those not meeting PTSD criteria (7.8) (*p* < 0.001). An analysis of these scores revealed that a combined score of 17 or more accurately identified >80% of children with a diagnosis of PTSD.

The CPDS is an instrument for assessing children's psychosocial distress and estimating the likelihood of their needing psychological treatment. The instrument was developed in and for non-Western emergencies as a primary screening tool in conflict-affected communities (54) The instrument includes an assessment of the child's traumatic distress, resilience components such as social support, and functioning through five items that are read to the child and then scored on a 3-point scale (0, 1, 2). The total score is in the range of 0–10.

The validity of the tool was tested on children from four countries: Burundi; Sudan; Sri Lanka; and Indonesia. We chose to use a cut-off of 8, indicating the presence of psychosocial distress, based on results from the Burundi sample used for the validation of the instrument, as this was the context closest to that of CAR. Because of its brevity and its ability to be administered by non-specialists, the CPDS can be used as a screening tool for large populations of children affected by conflict (55).

### Data analysis

To test whether there were statistically significant differences between the two groups in the sociodemographic characteristics, the chi-square or Fisher's exact test was performed, where appropriate. The independent samples *t*-test was used to test whether there was a difference in age, school level, gender, CPDS, and CRIES-8 between the two groups, for both 661 and 185 samples.

To test whether there were statistically significant differences between the ACF/KONO and G-TEP protocols, both pre- and post-treatment, we used a pairwise independent samples *t*-test.

In the sample of 185 children, to check whether there were statistically significant differences between the ACF/KONO and G-TEP groups before, after, and at follow-up, we used a multifactorial ANOVA for the CPDS score, as well as a pairwise independent samples *t*-test for the CRIES-8 score, as the pre-treatment samples did not meet the assumption of homogeneity of variance. The Bonferroni post-hoc test was used to analyze the differences between the ACF/KONO and G-TEP groups’ pre-treatment, post-treatment, and follow-up scores.

In the sample of 661 children, we tested whether there were statistically significant differences in the improvement of CRIES-8 and CPDS scores based on sociodemographic characteristics and the two protocols. We calculated the improvement score (the difference between pre-treatment and post-treatment scores) and compared it using multifactorial ANOVA, ensuring Levene's condition was met. Otherwise, the Kruskal–Wallis non-parametric ANOVA were used. We chose to split the age groups into two categories, with 12 years being the median age: the group of children aged 12 years or older comprised 50.3% of the sample, and the group aged 11 years or younger comprised 49.7% of the sample.

*p* < 0.05 was considered to be statistically significant. The statistical analysis was performed using Jamovi statistical software package version 2.3.28.0 [The Jamovi project (2021). Jamovi. Version 2.2, retrieved from https://www.jamovi.org].

## Results

**Figure 2:**
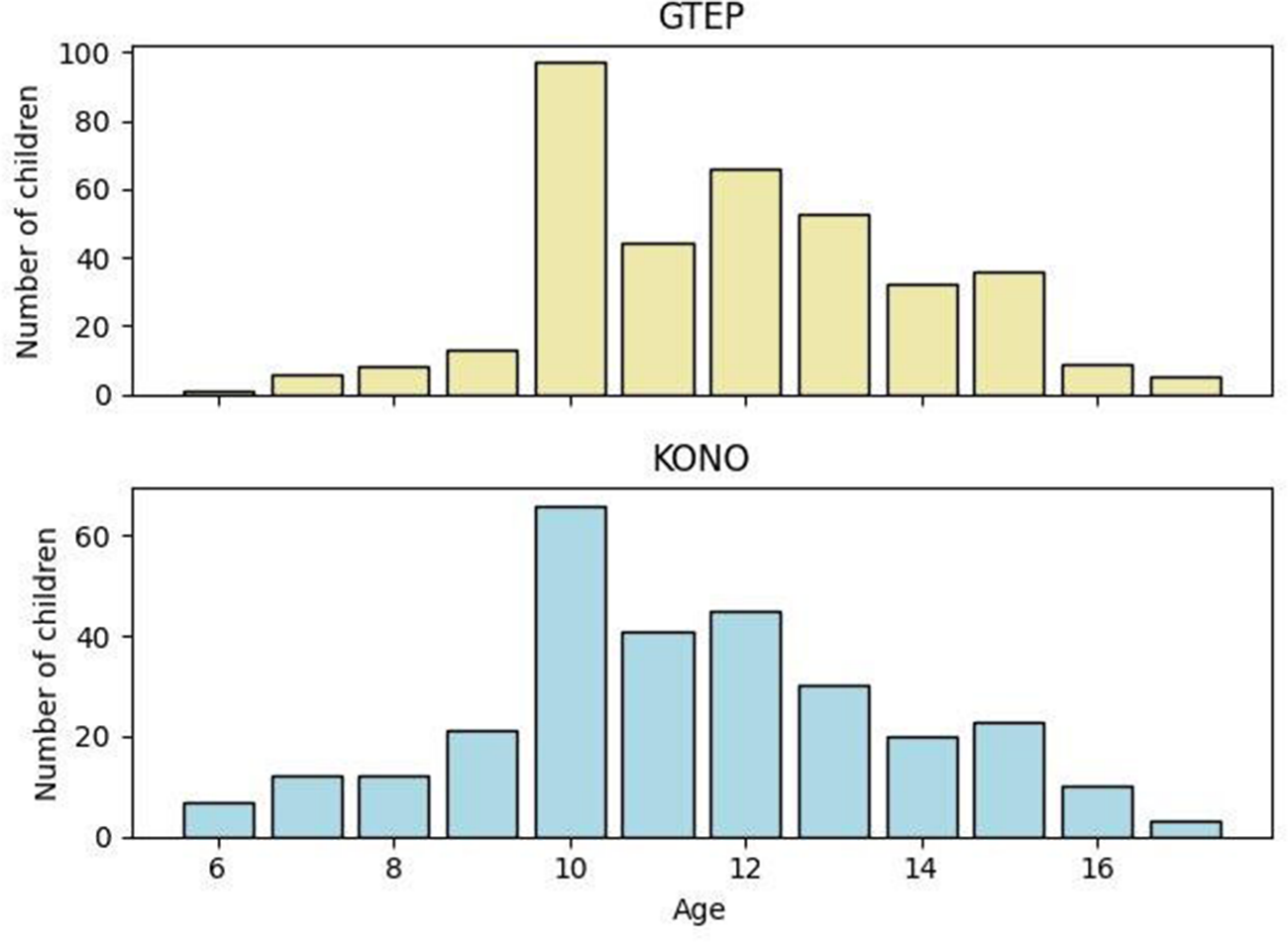
Age distribution in the two protocols.

The values of the sociodemographic variables between ACF/KONO and G-TEP participants are presented in Table 1. There was no statistically significant difference regarding gender (χ^2^ = 0.996; *p* = 0.318) and level of education (χ^2^ = 2.40; *p* = 0.493). There was a significant difference in relation to age (*t* = −1.0930; *p* = 0.861), but the distribution between the two groups was not homogeneous (Figure 2). Participants in the G-TEP protocol were older than those in the ACF/KONO protocol.

**Table 1:** Sociodemographic data.

Sociodemographic characteristics	ACF/KONO (*n* = 290)	G-TEP (*n* = 371)	Analysis		
			χ^2^	*df*	*p*
Gender, *n* (%)			0.996	1	0.318
Boys	145 (50%)	171 (46%)			
Girls	145 (50%)	200 (54%)			
Education, *n* (%)			2.40	3	0.493
None	14 (4.8%)	25 (6.73%)			
Primary	276 (95.1%)	342 (92.1%)			
Secondary	0 (0%)	1 (0.2%)			
			*t*	*df*	*p*
Age, M (SD)	11.3 (2.40)	11.8 (2.10)	2.87	658	0.004

M, mean; SD, standard deviation.

The CRIES-8 and CPDS scores are shown in Table 2.

**Table 2:** Mean (8SD) scores in pre-treatment and post-treatment tests in the two protocols.

	Pre		Post	
	ACF/KONO	G-TEP	ACF/KONO	G-TEP
Measure (*n* = 661)				
CRIES-8, M (SD)	27.75 (4.70)	27.75 (5.42)	10.90 (4.90)	10.64 (4.91)
CPDS, M (SD)	7.80 (1.15)	7.72 (1.15)	3.62 (1.46)	3.32 (1.24)

M, mean; SD, standard deviation.

The sociodemographic profile of children who completed the study and those who dropped out showed no difference in terms of age (*t* = −1.39 *p* = 0.164), gender (χ² = 0.893 *p* = 0.345), and education (χ² = 2.30 *p* = 0.513).

A comparison of the psychological profile at the assessment in the pre-treatment phase of children who completed the study with those who dropped out showed no difference in CPDS scores (*t* = −1.51; *p* = 0.132) and a significant difference for CRIES-8 scores (*t* = 5.73; *p* < 0.001), indicating lower scores for children who interrupted the intervention.

At the assessment in the pre-treatment phase, the mean CPDS scores of participants in the ACF/KONO group and the G-TEP group did not differ (*t* = −0.88; *p* = 0.378). Nor did the two groups differ in CRIES-8 scores (*t* = −0.002; *p* = 0.998). The mean CPDS scores of both groups showed a level of general distress below the cut-off point of 8. The mean CRIES-8 scores of participants in both groups were well above the cut-off point of 17, underlining high levels of PTSD.

### Efficacy of ACF/KONO and G-TEP protocols

The Student's paired-samples *t*-test for ACF/KONO participants showed a significant effect, with reduced scores on the CRIES-8 (*t* = 44.8; *p* < 0.001, effect size = 2.63) and CPDS (*t* = 38.2; *p* < 0.001, effect size = 2.24). The mean post-treatment CRIES-8 score was below the clinical threshold for PTSD (<17), with 11% of children showing scores above or equal to the cut-off. The mean post-treatment CPDS score remained below the clinical cut-off (<8), with 2% of children showing scores above or equal to the cut-off.

Student's paired-samples *t*-test for the G-TEP protocol showed a significant effect, with reduced scores on the CRIES-8 (*t* = 49.2; *p* < 0.001, effect size = 2.55) and CPDS (*t* = 57.2; *p* < 0.001, effect size = 2.97). The mean post-treatment CRIES-8 score was below the clinical cut-off for PTSD (<17); 10% of children showed scores above or equal to the cut-off. The mean post-treatment CPDS score remained below the clinical cut-off (<8); 1% of children showed scores above or equal to the cut-off.

**Figure 3:**
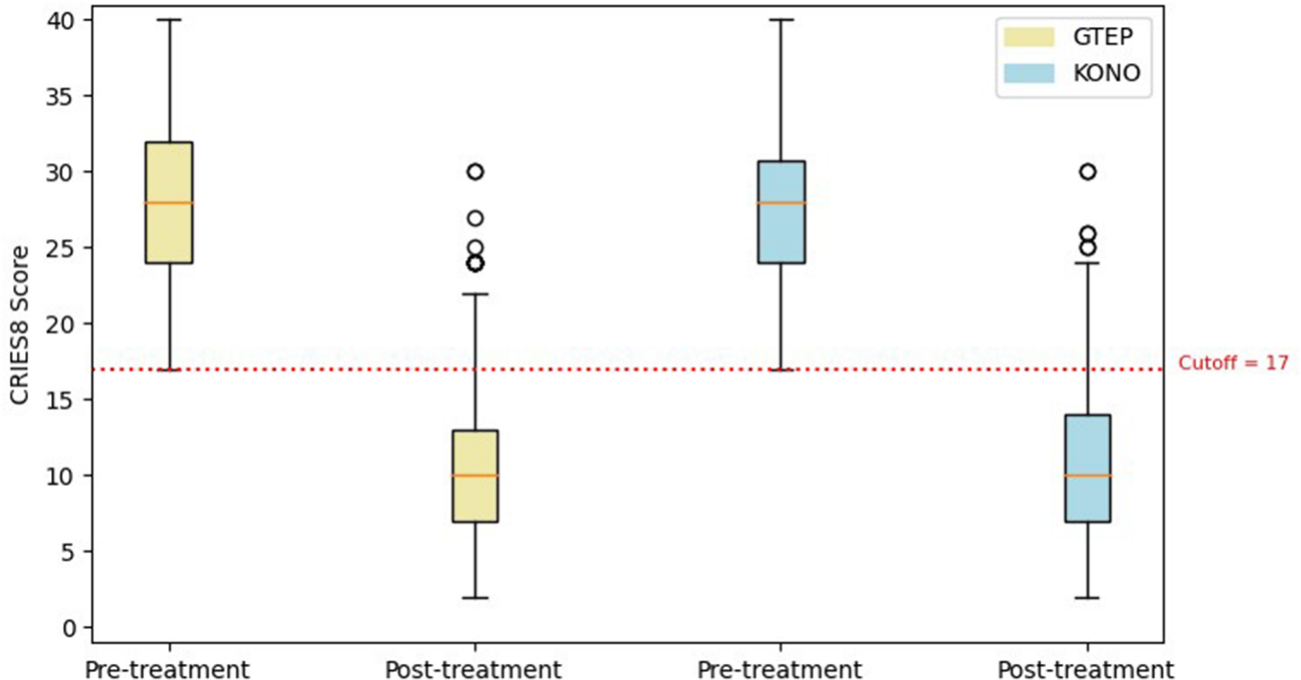
Box plot displaying differences in pre- and post-treatment CRIES-8 scores of the two protocols. The circle in the box plot represents extreme values.

**Figure 4:**
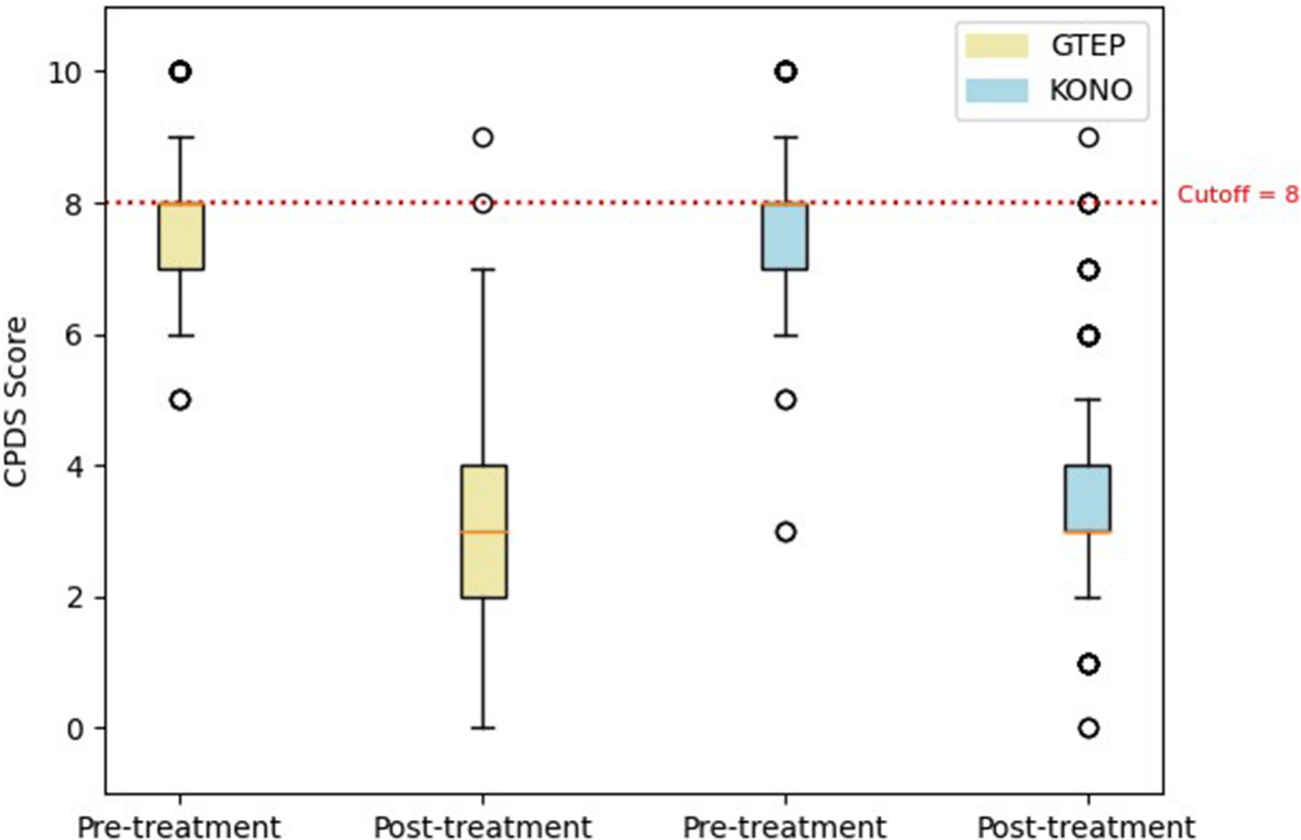
Box plot displaying differences in pre- and post-treatment CPDS scores of the two protocols. The circle in the box plot represents extreme values.

The Student's *t*-test comparing the two groups ACF/KONO and G-TEP showed no significant difference between the two types of treatment between the pre- and post-treatment CRIES-8 scores (*t* = 1.744; *p* = 0.514, effect size = 0.040) (see Figure 3) and CPDS scores (*t* = 1.688; *p* = 0.092, effect size = 0.323) (see Figure 4).

At the post-treatment assessment, the mean scores of participants in the ACF/KONO group and the G-TEP group did not differ for the CRIES-8 (*t* = −0.88; *p* = 0.005) but they were statistically different for the CPDS (*t* = −0.687; *p* = 0.492), suggesting slightly lower values for the G-TEP protocol (see Table 2).

### Relation with sociodemographic variables

For the CRIES-8 betterment score, there was a statistically significant interaction between gender and protocol (*F* = 5.522, *p* = 0.019), showing a larger mean betterment score on boys treated with G-TEP (see Table 3), whereas ACF/KONO demonstrated a larger mean betterment score on girls.

**Table 3:** Gender and age mean (and SD) of CRIES-8 and CPDS betterment score according to the protocol.

			G-TEP	KONO
CRIES-8	Gender	Girls, M (SD)	16.6 (6.58)	17.5 (5.86)
		Boys, M (SD)	17.8 (6.81)	16.2 (6.86)
	Age	≥12 years, M (SD)	18 (6.56)	16.4 (6.48)
		<12 years, M (SD)	16.1 (6.73)	17.2 (6.33)
CPDS	Gender	Girls, M (SD)	4.42 (1.44)	4.21 (2.01)
		Boys, M (SD)	4.37 (1.54)	4.14 (1.70)
	Age	≥12 years, M (SD)	4.50 (1.39)	4.21 (1.75)
		<12 years, M (SD)	4.28 (1.58)	4.15 (1.95)

M, mean; SD, standard deviation.

There was a statistically significant interaction between age and protocol (*F* = 7.238, *p* = 0.007), showing a larger mean betterment score on children aged 12 years and older treated with G-TEP (see Table 3), whereas ACF/KONO demonstrated a larger mean betterment score on children aged below 12 years.

For the CPDS betterment score, there was no statistically significant difference between gender and protocol (χ² = 1.90, *p* = 0.594). There was no statistically significant difference between age and protocol (χ² = 4.21, *p* = 0.378).

The effect of education on the betterment score was not analyzed, as 93.8% of children have a primary school education (see Table 1), meaning the other groups were not large enough to lead to a statistically significant analysis.

### Efficacy of the two protocols after 5 months

Due to the security situation, which prevented access to certain areas, we were able to return to two intervention areas 5 months after the end of treatment to re-measure the CPDS and CRIES-8 scales in 185 children. Details of the sociodemographic variables for this sample are given in Table 4.

**Table 4:** Sociodemographic data of the sample (*n* = 185).

Sociodemographic characteristics	ACF/KONO (*n* = 90)	G-TEP (*n* = 95)	Analysis		
			χ^2^	*df*	*p*
Gender, *n* (%)			1.19	*1*	0.276
Boys	47 (52%)	42 (44.2%)			
Girls	43 (47%)	53 (55.7%)			
Education *n* (%)			3.15	2	0.207
None	3 (3.3%)	9 (9.4%)			
Primary	87 (96%)	86 (90.5%)			
			*t*	*df*	*p*
Age, M (SD)	11.7 (2.07)	11.9 (1.90)	0.638	183	0.524

M, mean; SD, standard deviation.

In the sample of 185 children with the three measures, pre-treatment, post-treatment, and follow-up after 5 months, a multifactor ANOVA was used to evaluate the CDPS and the CRIES-8 scores over time (see Table 5).

**Table 5:** CPDS and CRIES-8 mean scores (SD) for a sample of 185 children with pre-treatment, post-treatment, and follow-up measurements.

	Pre		Post		Follow-up/5 months	
	ACF/KONO	G-TEP	ACF/KONO	G-TEP	ACF/KONO	G-TEP
Measure (*n* = 185)						
CRIES-8, M (SD)	28.6 (4.03)	25.8 (4.90)	11.6 (5.88)	11.4 (5.41)	7.53 (4.15)	7.84 (5.34)
CPDS, M (SD)	7.66 (1.30)	7.78 (1.08)	3.35 (1.63)	3.53 (1.26)	2.89 (1.77)	2.43 (1.51)

M, mean; SD, standard deviation.

We compared the pre-evaluation profile between children who were re-evaluated for follow-up 5 months after the end of the intervention and those who were lost to follow-up. No significant differences were found for the following sociodemographic variables: age (*t* = 1.016; *p* = 0.310), gender (χ² = 0.009; *p* = 0.923), and education (χ² = 0.883; *p* = 0.830). No difference was found for scores of CDPS (*t* = −0.459; *p* = 0.646) and CRIES-8 (*t* = −1.911; *p* = 0.056).

**Figure 5:**
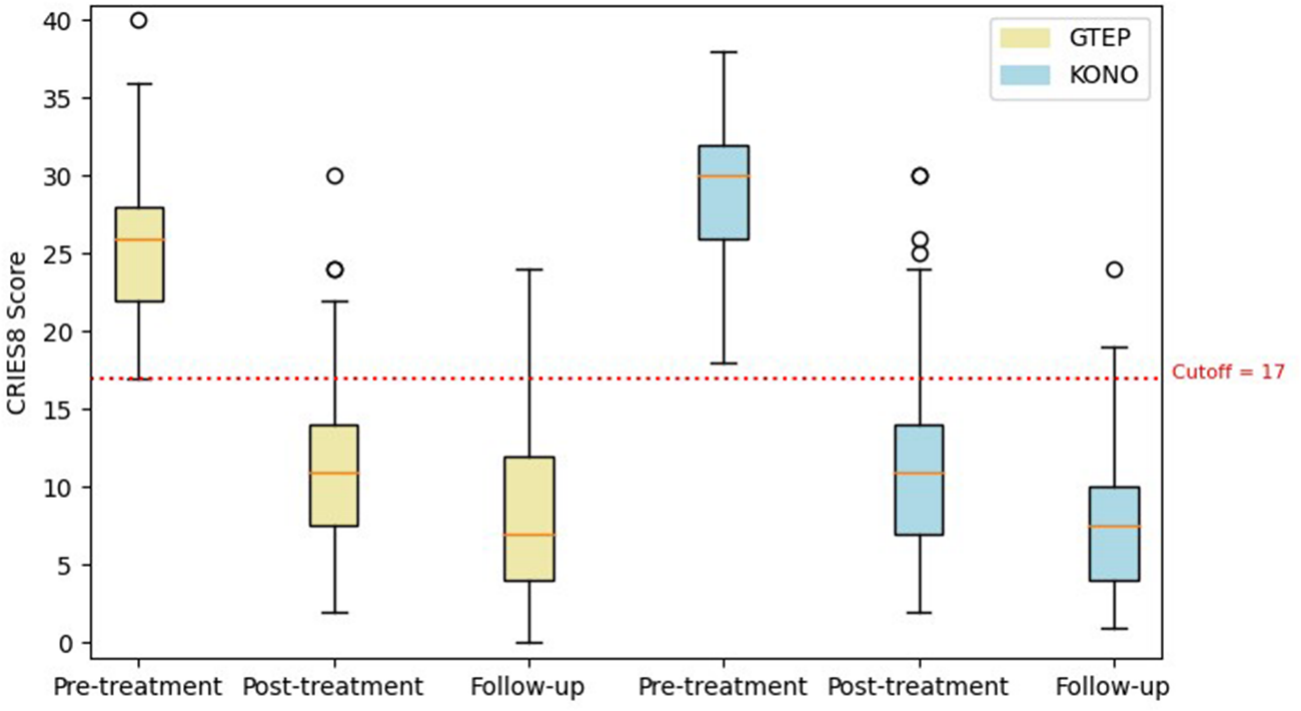
Box plot displaying differences in pre- and post-treatment CRIES-8 scores and follow-up of the two protocols. The circle in the box plot represents extreme values.

Repeated measures analysis on CRIES-8 (Figure 5) revealed a significant group effect (*F* = 890.73; *p* < 0.001). CRIES-8 scores were significantly different before treatment (difference = −2.756, SE = 0.662; *p* < 0.001), with higher values in the ACF/KONO group (see Table 4). There was no significant difference in mean scores between participants in the two protocols for the CRIES-8 after treatment (difference = −0.189; SE = 0.830; *p* = 1.000) and after follow-up (difference = 0.309, SE = 0.706; *p* = 0.998).

The analysis revealed a significant time effect (*F* = 5.64; *p* = 0.004). A Bonferroni *post-hoc* test of the time effect for ACF/KONO protocol of CRIES-8 scores revealed a statistically significant difference between the pre- and post-treatment scores (difference = 16.967, SE = 0.714; *p* < 0.01), between the pre-treatment and follow-up scores (difference = 21.022, SE = 0.681; *p* < 0.01) as well as between the post-treatment and follow-up scores (difference = 4.056, SE = 0710; *p* < 0.01).

The Bonferroni *post-hoc* test of the time effect for G-TEP protocol of CRIES-8 scores revealed a statistically significant difference between the pre- and post-treatment scores (difference = 14.400, SE = 0.695; *p* < 0.001), between the pre-treatment and follow-up scores (difference = 17.958, SE = 0.663; *p* < 0.001), and between the mean post-treatment and follow-up scores (difference = 4.056, SE = 0.710; *p* < 0.001).

Group by time interaction was not significant for CRIES-8 scores (*F* = 3.47; *p* = 0.064).

**Figure 6:**
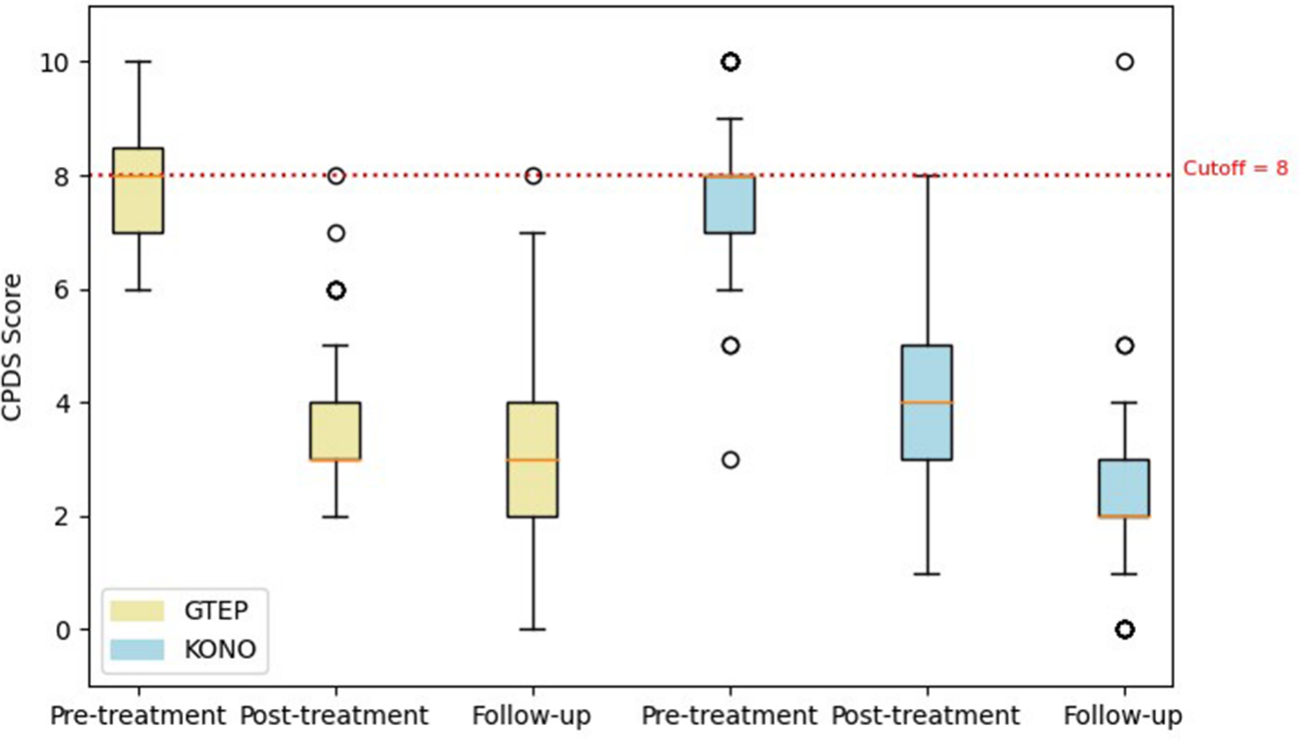
Box plot displaying differences in pre- and post-treatment CPDS scores and follow-up of the two protocols. The circle in the box plot represents extreme values.

Repeated measures analysis on CPDS scores (Figure 6) revealed no significant group effect (*F* = 0.114; *p* = 0.736): there was no significant difference in CPDS mean scores between participants in the ACF/KONO and G-TEP protocols, before treatment (difference = 0.123, SE = 0.176; *p* = 0.981), after treatment (difference = 0.451, SE = 0.214; *p* = 0.285), and at follow-up (difference = 0.461, SE = 0.242; *p* = 0.403).

The analysis revealed a significant time effect (*F* = 681.35; *p* < 0.001).

The Bonferroni *post-hoc* test of the time effect for ACF/KONO protocol of CPDS scores revealed a statistically significant difference between the pre-treatment and post-treatment scores (difference = 3.678, SE = 0.194; *p* < 0.01), a statistically significant difference between the pre-treatment and follow-up scores (difference = 5.222, SE = 0.207; *p* < 0.01) as well as a statistically significant difference between post-treatment and follow-up scores (difference = 1.544, SE = 0.218; *p* < 0.01).

The Bonferroni post-hoc test of the time effect for EMDR/G-TEP protocol of CPDS scores revealed a statistically significant difference between the pre-treatment and post-treatment scores (difference = 4.253, SE = 0.189; *p* < 0.001) and between the pre-treatment and follow-up scores (difference = 4.484, SE = 0.201; *p* < 0.001). There was no significant difference between the mean post-treatment and follow-up scores (difference = 0.632, SE = 0.212; *p* = 0.038).

Group by time interaction was significant (F = 5.13; *p* = 0.006).

In both protocols, mean scores after treatment and at follow-up remained below the clinical cut-off for both CPDS (<8) and CRIES-8 (<17). The percentages of children with scores above or equal to the clinical cut-off after treatment and at follow-up are detailed in Table 6.

**Table 6:** Number (%) of children above the cut-off scores (sample *n* = 185).

	CRIES-8				CPDS			
	G-TEP		ACF/KONO		G-TEP		ACF/KONO	
	*n*	%	*n*	%	*n*	%	*n*	%
Pre-treatment	95	100%	90	100%	61	64%	52	58%
Post-treatment	14	15%	16	18%	1	1%	3	3%
Follow-up	5	5%	2	2%	1	1%	1	1%

### Summary of the results

In total, 661 children (290 for ACF/KONO and 371 for G-TEP) benefited from all treatment sessions.

ACF/KONO and G-TEP participants showed no statistically significant difference on sociodemographic variables, except for age: participants in the G-TEP protocol were older than those in the ACF/KONO protocol. At the pre-treatment assessment, the mean CPDS scores did not differ between the groups (*t* = −0.88; *p* = 0.378), nor did the two groups differ in CRIES-8 scores (*t* = −0.002; *p* = 0.998). The mean CPDS scores of both groups showed a level of general distress and the mean CRIES-8 scores in both groups showed high levels of PTSD.

After the intervention, ACF/KONO participants showed a significant reduction on CRIES-8 (*t* = 44.8; *p* < 0.001, effect size = 2.63) and CPDS (*t* = 38.2; *p* < 0.001, effect size = 2.24). Participants in the G-TEP protocol showed a significant effect, with reduced scores on the CRIES-8 (*t* = 49.2; *p* < 0.001, effect size = 2.55) and CPDS (*t* = 57.2; *p* < 0.001, effect size = 2.97). Student's *t*-test comparing the ACF/KONO and G-TEP groups showed no significant difference between the pre- and post-treatment CRIES-8 scores (*t* = 1.744; *p* = 0.514, effect size = 0.040) and CPDS scores (*t* = 1.688; *p* = 0.092, effect size = 0.323). The results for CRIES-8 showed a statistically significant interaction between gender and the type of protocol used, showing a bigger improvement in boys treated with G-TEP, unlike the ACF/KONO protocol, showing a bigger improvement in girls.

There was a statistically significant interaction between age and protocol type: children aged 12 years and over showed a bigger improvement with the G-TEP protocol, while children aged under 12 years showed a bigger improvement with the ACF/KONO protocol.

For the CPDS improvement score, there was no statistically significant difference between the gender and age variables and the protocols.

An analysis of the follow-up data for the 185 children showed that the effects of both protocols are maintained over time, with mean scores before treatment and at follow-up below the clinical cut-off for both CPDS (<8) and CRIES-8 (<17).

## Discussion

The aim of this study was to evaluate whether the G-TEP protocol could be an alternative to the ACF/KONO protocol, which has been used for several years by the NGO Action contre la Faim in humanitarian emergency contexts. This need emerged from the fact that few proposals for clinical management and reduction of traumatic symptoms are offered to children in situations of exposure to mass trauma. Literature reviews on mental health and psychosocial support interventions for children in low- and middle-income countries, particularly in conflict situations, highlight a strong need for structured therapeutic protocols that can be deployed on a large scale (56, 57).

One difficulty that limits psychological support interventions for children is the lack of professionals trained in the management of children's trauma, particularly in developing countries where there are few, if any, training curricula on the subject. To meet the significant psychological support needs of children affected by crisis situations, such as conflicts, natural disasters, epidemics, etc., it is necessary to be able to offer psychological interventions carried out by duly trained and supervised mental health paraprofessionals (58, 59).

The ACF/KONO protocol has been designed to enable paraprofessionals to set up a treatment program to reduce symptoms of trauma in children. Until the current project, ACF had never used the G-TEP protocol on children. To our knowledge, very few studies have been carried out on the efficacy of G-TEP for children, and no publications are currently available. Our study was based on data collected within an ACF psychological support project, which enabled a comparison between the ACF/KONO protocol and the G-TEP protocol. The project was complicated by the specific conditions of the humanitarian field, where it is often difficult to guarantee a certain rigor in the implementation of interventions and in data collection (60).

In the specific case of the Central African Republic, the project was carried out in an area of ongoing conflict, which prevented regular access to the intervention zones by either the team or the participating children. Despite these difficulties, which prevented the 16% of children initially included in the project from attending regularly, participation was remarkably high. A total of 661 children attended the five sessions planned by each of the two therapeutic systems. This program was the first to use two post-traumatic symptom reduction protocols with such a large sample in a context of insecurity and conflict. This remarkable participation in psychological sessions in a context of extreme precariousness and danger can also be interpreted as an important indicator of the interest and priority given to such care for children, by families and communities, even though the program did not provide any benefits or financial advantages. Another objective of the study was to check whether the treatment results were maintained over time. Indeed, the activities proposed in this program were carried out very soon after exposure to critical incidents in a context of chronic and repetitive violence and insecurity. Early interventions in acute phases aim to both treat post-trauma symptoms and prevent the development of chronic PTSD (61) as it is important to be able to offer affected populations both immediate relief from their traumatic suffering and to limit the installation and chronicization of traumatic symptoms. It was impossible to contact again all the children who had participated in the program for a follow-up reassessment after the end of treatment. However, it was possible to reassess the wellbeing and level of traumatic symptoms of 185 children 5 months after the end of treatment. The data collected were sufficiently substantial to enable us to provide answers on the long-term efficacy of these two protocols.

With both the ACF/KONO and G-TEP protocols, the CDPS and CRIES-8 scores decreased significantly after treatment. Very few children showed symptoms of trauma after treatment with either protocol. Our results show that both protocols are effective in improving wellbeing and treating trauma in children. There was a difference in post-treatment CDPS scores, which were significantly better using the G-TEP protocol.

Analyses carried out on the sample of children who received an assessment 5 months after treatment showed that the effects of both interventions are maintained, and even continue to decrease significantly, over time. These results confirm that these early interventions may also have a role to play in preventing the chronicization of post-traumatic symptoms.

The results show no significant difference between the two protocols in terms of either improvement in general wellbeing or reduction in traumatic symptoms. The only difference was in the effects between post-evaluation and follow-up. The G-TEP protocol did not show a significant difference in CPDS scores. This small difference does not suggest a significant difference in the efficacy of the two protocols. At the pre-treatment assessment, the children showed very high scores on the CRIES-8, indicating a significant level of traumatic symptoms. However, the CPDS scores were below the cut-off score of 8, although they were not far from it (ACF/KONO: M = 7.80, SD = 1.15; G-TEP: M = 7.72, SD = 1.15). This may be due to the choice of cut-off score, which may need to be adjusted for the CAR target population. The CPDS validation paper shows that the tool can be used in many different cultural contexts for the assessment of non-specific psychosocial distress, as the constituent elements of psychosocial distress may be similar from one context to another, but the specific way in which they manifest themselves could be different. The use of a different cut-off score may be more relevant in CAR and may be the subject of further research, as the contextual validity of validation tools is strongly recommended (62, 63).

The analysis of sociodemographic data in relation to the two protocols showed that girls performed better with the ACF/KONO protocol, and that older children performed better with the G-TEP protocol. It may be premature to advance generalizable conclusions based on this study; however, we can propose some hypotheses.

The ACF/KONO protocol is a trauma-focused narrative protocol, and the traumatic experience is verbalized and thus shared within the group. The G-TEP protocol, on the other hand, proposes individual work within a group (each child works on his or her own sheet of paper). This difference in the nature of the protocol could explain why the data show a gender difference in effectiveness. Boys may be more comfortable with a more individual approach, where the expression of their emotional experience is more reduced. Girls may be more comfortable recognizing and expressing distress in a group setting. Furthermore, in the G-TEP protocol, the child, guided by the paraprofessional, processes his trauma and connects to his past, present, and future resources, whereas the ACF/KONO protocol, through the group and narrative, also encourages the child to rely on others, on external resources, to overcome his difficulties. The way in which people cope with and manage their difficulties in order to get better may also be gendered in Central African culture and could explain the differences observed.

The nature of the protocol could also explain the age-related difference in score improvement: the ACF/KONO protocol involves the use of picture boards representing the life of a hippopotamus that faces difficulties and then copes with them with the help of those around it and the reinforcement of his own internal resources. Although this protocol has proved effective in several contexts for implementing mental health and psychosocial support projects, the images used seem to be better suited to younger children. It might be appropriate to adapt the images and protagonists of the protocol for an adolescent audience, to facilitate projection and identification. More in-depth studies could focus more on children's appreciation of the two protocols and their feedback about the protocols, emotional sharing and traumatic experiences within the group, to test the hypotheses proposed here. They could also provide new criteria for arbitration between the two protocols according to child populations and contexts.

With minimal differences, both protocols reduced children's symptoms of trauma and were therefore contextually appropriate for use with children in humanitarian crisis situations.

The results presented in this study confirm the findings of previous studies on the use of the ACF/KONO protocol (43), as well as the unpublished results obtained in the NGO's programs set up in several humanitarian contexts.

As for the G-TEP protocol, given the lack of publication on its use with children, we can base our observations on results obtained with adults. Our results confirm those of studies describing the use of G-TEP with an adult population (25). In addition, the results of this study are similar to the results of studies on the comparison of treatment between G-TEP and a CBT protocol with adults in Iraq (49) and the Central African Republic^1^, in which post-traumatic symptoms decreased significantly with each protocol; however, there was no difference in efficacy between the two protocols. In adults in CAR, as in our study with children, the G-TEP results were greater in men than in women, perhaps highlighting a gender-related cultural difference depending on the protocol.

Both protocols were conducted by paraprofessionals under the supervision of clinical psychologists, remotely and in the field. This shows that both types of treatment are feasible in the real-life conditions of a humanitarian context and lack of specialized personnel. This confirms that it is possible to fill gaps in mental health and psychosocial support using paraprofessionals (64), with technical supervision provided by mental health specialists (32, 65, 66).

The use of the G-TEP protocol was tested to answer clinical questions following the use of the ACF/KONO protocol. The data showed that both protocols were equally effective in reducing children’s traumatic symptoms, both at the end of treatment and 5 months later.

The reduction in symptoms of trauma is due to the psychic elaboration mechanisms underlying each protocol, which have points in common, but also important differences.

Among the points in common, there is the fact of working on individual psychic resources to help children contextualize the traumatic event and put it into perspective in a more appropriate personal narrative so that the traumatic traces are less painful, and the prospect of the future can be envisaged. In both protocols, drawing is used to externalize internal experiences, which take shape through a trace on paper. Physical and visual anchoring helps to set traumatic experiences and personal resources in movement, breaking out of the immobility of the traumatic experience.

The group helps break out of the isolation characteristic of traumatic symptoms and to regain the security needed to process the traumatic experience.

We observed two differences in particular that seem to differentiate the protocols, the first being linked to the structure of the two treatment devices.

One of the reasons for testing G-TEP was to find a protocol that could be as effective as the ACF/KONO protocol, but with greater flexibility for use in volatile contexts in conflict situations. In fact, each G-TEP session is identical, enabling participants to work on traumatic events. Its effectiveness has been demonstrated with a limited number of sessions with adults (from the very first session). The ACF/KONO protocol, on the other hand, has a narrative structure that requires all five sessions to be completed. Our results confirm that both protocols are equally effective over five sessions; however, our data do not allow us to know whether the results obtained are faster with one protocol than the other. To answer this question, it would be interesting to measure distress and post-traumatic stress at each session, or at least in the middle of the treatment, to see whether G-TEP would be more effective more quickly.

The second difference between the protocols concerns the fact that the ACF/KONO protocol offers a space for talking about difficult and painful moments, which is not the case in the G-TEP protocol, where group sharing is focused on positive aspects and projection into the future only. This point should also be considered in the vicarious trauma of the psychosocial workers who lead the sessions.

Paraprofessionals working in conflict zones are often people who are themselves affected by the situation in their own country, and who come to the aid of their compatriots (67).

The impact of each protocol on the teams could be another key factor to consider when choosing protocols. G-TEP may be more protective of traumatic transmission since the children do not share their traumatic experiences verbally (68). In this study, we were unable to compare vicarious trauma and/or the impact of the protocols on the teams, as the same teams worked on both protocols. However, it would be useful to conduct a study in the future that would assess the consequences of the type of protocol chosen on teams’ emotional wellbeing, vicarious trauma, and other variables, such as burnout, motivation, and compassion fatigue.

The choice of protocol could be guided by the composition of the groups, given the observed differences in age and gender, as well as the appetence of paraprofessionals or children benefiting from the therapeutic device.

Although further studies are needed to support the results presented in this paper, the data are very encouraging about the possibility of using these two protocols in humanitarian contexts and by paraprofessionals.

In conclusion, this retrospective study confirms the validity of the strategy for scaling up this EMDR-adapted protocol for children. It shows that the G-TEP protocol conducted by paraprofessionals reduces post-traumatic symptoms in children aged 6–17 years as effectively and significantly as the ACF/KONO protocol over five sessions. These results were maintained 5 months after the end of treatment, even though the children were still living in a context of chronic insecurity and violence. These treatments were carried out by paraprofessionals supervised by psychologists in a humanitarian program that was as close as possible to the realities of emergencies and enabled over 661 children to be treated over a 10-month period. They open up new perspectives on the possibility of offering children effective approaches to treating their post-traumatic symptoms by deploying large-scale programs with non-specialized but well-trained and supervised staff.

## Data Availability

The raw data supporting the conclusions of this article will be made available by the authors, without undue reservation.

## Footnotes

^1^Dozio E, Bizouerne C, Lassalle N, Farrell D. Comparaison de protocoles de groupe EMDR et Thérapie Cognitivo-Comportementale (TCC) pour traiter les traumatismes en contexte humanitaire mis en place par des paraprofessionnels. (in press).
